# Serotypes and Clonal Diversity of *Streptococcus pneumoniae* Causing Invasive Disease in the Era of PCV13 in Catalonia, Spain

**DOI:** 10.1371/journal.pone.0151125

**Published:** 2016-03-08

**Authors:** Eva del Amo, Cristina Esteva, Susanna Hernandez-Bou, Carmen Galles, Marian Navarro, Goretti Sauca, Alvaro Diaz, Paula Gassiot, Carmina Marti, Nieves Larrosa, Pilar Ciruela, Mireia Jane, Raquel Sá-Leão, Carmen Muñoz-Almagro, Catalan Study Group of Invasive Pneumococcal Disease

**Affiliations:** 1 Department of Molecular Microbiology, Hospital Sant Joan de Deu, Esplugues de Llobregat, Spain; 2 Department of Paediatrics, Hospital Sant Joan de Deu and University of Barcelona, Esplugues de Llobregat, Spain; 3 Department of Microbiology, Hospital Sant Jaume, Calella, Spain; 4 Department of Microbiology, Hospital de Vic, Vic. Spain; 5 Department of Microbiology, Hospital de Mataró, Mataró, Spain; 6 Department of Microbiology, Hospital de Nens, Barcelona, Spain; 7 Department of Microbiology, Hospital de Figueres, Figueres, Spain; 8 Department of Microbiology, Hospital General de Granollers, Granollers, Spain; 9 Department of Microbiology, Hospital Vall d’Hebron, Barcelona, Spain; 10 Public Health Agency, Government of Catalonia, Barcelona, Spain; 11 Laboratory of Molecular Microbiology of Human Pathogens, Instituto de Tecnologia Química e Biológica, Universidade Nova de Lisboa, Oeiras, Portugal; Rockefeller University, UNITED STATES

## Abstract

The aim of this study was to study the serotypes and clonal diversity of pneumococci causing invasive pneumococcal disease in Catalonia, Spain, in the era of 13-valent pneumococcal conjugate vaccine (PCV13). In our region, this vaccine is only available in the private market and it is estimated a PCV13 vaccine coverage around 55% in children. A total of 1551 pneumococcal invasive isolates received between 2010 and 2013 in the Molecular Microbiology Department at Hospital Sant Joan de Déu, Barcelona, were included. Fifty-two serotypes and 249 clonal types—defined by MLST—were identified. The most common serotypes were serotype 1 (*n* = 182; 11.7%), 3 (*n* = 145; 9.3%), 19A (*n* = 137; 8.8%) and 7F (*n* = 122; 7.9%). Serotype 14 was the third most frequent serotype in children < 2 years (15 of 159 isolates). PCV7 serotypes maintained their proportion along the period of study, 16.6% in 2010 to 13.4% in 2013, whereas there was a significant proportional decrease in PCV13 serotypes, 65.3% in 2010 to 48.9% in 2013 (p<0.01). This decrease was mainly attributable to serotypes 19A and 7F. Serotype 12F achieved the third position in 2013 (n = 22, 6.4%). The most frequent clonal types found were ST306 (*n* = 154, 9.9%), ST191 (*n* = 111, 7.2%), ST989 (*n* = 85, 5.5%) and ST180 (*n* = 80, 5.2%). Despite their decrease, PCV13 serotypes continue to be a major cause of disease in Spain. These results emphasize the need for complete PCV13 vaccination.

## Introduction

*Streptococcus pneumoniae* is a major cause of morbidity and mortality worldwide, especially among young children and the elderly. This pathogen colonizes the nasopharynx as part of the normal flora in children [1] and is the leading bacterial cause of pneumonia, sepsis and meningitis [2]. During asymptomatic nasopharyngeal colonization stages, *S*. *pneumoniae* interplays with the host immune system through multiple and complex interactions. Imbalance due to host factors and microbiological factors are related with the propensity to cause serious invasive disease [3]. The capsule of pneumococcus is its main virulence factor. There are over 97 known serotypes but not all of them have the same capacity to cause invasive pneumococcal disease (IPD) [4].

Despite the high effectiveness reported for PCV7 worldwide [5–8], the lower vaccine serotype coverage found in some countries compared to the USA [9] made necessary the development of two new conjugate vaccines, PCV10 and PCV13, which included a larger number of serotypes. PCV13 vaccine includes serotypes 1, 3, and 5 that were common in Europe, Asia, and Africa, and also serotype 6A, 7F and 19A. The majority of these serotypes had emerged after the implementation of PCV7 in our region [10].

Of note, although the Vaccination Advisory Committee of the Spanish Association of Pediatrics has recommended the routine administration of conjugate pneumocccal vaccines (PCV7 since 2001–2010 and currently PCV13) these vaccines have not been financed by the Catalan Public Health System and they have been only available in the private market. In our reference area, we estimated a PCV7 vaccination coverage of 47% in 2007 [10] and a PCV13 vaccine coverage of 55% in 2013 [11].

Although differences in the capsular type are the major determinant of the invasive disease potential of a strain, several studies have suggested that differences in both the invasive disease potential and the ability to cause disease are also influenced by the genetic background [12–14].

The aim of this study was to determine the pneumococcal serotypes and clonal types causing IPD in Catalonia, Spain, in the era of PCV13.

## Materials and Methods

This research was approved by the Ethics and Clinical Research Committee from Hospital Sant Joan de Déu. No consent was asked to participants because the data were analyzed anonymously.

This was a prospective study which included all pneumococcal invasive isolates, adults and children, received between 2010 and 2013 from 26 health centers in Catalonia that were characterized by the Molecular Microbiology Department at University Hospital Sant Joan de Déu in Barcelona, Spain. In 2009, this laboratory was designated by the government of Catalonia, Spain, for molecular surveillance of invasive pneumococcal disease. In Catalonia, with a population of around 7 million and 1.4 million persons aged 18 years or younger, these 26 health centres captured 40.1% of all hospital admissions during 2012 [15].

On December 9, 2009, the European Medicine Agency (EMA) authorized the commercialization of Pneumococcal Conjugate Vaccine 13 (PCV13) in Europe [16]. Although in Catalonia the pneumococcal conjugate vaccine has not yet been incorporated into the official vaccination calendar, PCV13 was included in the schedule recommended by the Spanish Association of Paediatrics in 2010 [17]. In fact, many paediatricians recommend this vaccine in their private practices, and vaccination coverage was estimated at 55.1% for PCV13, 12.5% for PCV7 and 1.4% for PCV10 vaccine in 2013 [11].

IPD was defined as the presence of clinical findings of infection together with isolation by culture of *S*. *pneumoniae* in sterile fluid samples (blood, cerebrospinal fluid or any other sterile fluid).

### Microbiological identification and antimicrobial susceptibility

All pneumococcal invasive isolates were cultured on blood agar plates (Columbia agar supplemented with 5% sheep blood; bioMérieux) and were identified in the different health centers by standard microbiological methods including optochin sensitivity test and an antigenic test. The minimum inhibitory concentrations (MIC) to antibiotics were determined with the agar dilution technique. Antibiotic susceptibilities were defined according to the breakpoints of the European Committee on Antimicrobial Susceptibility Testing (EUCAST) [18]. Multidrug resistance was defined as non-susceptibility to three or more antimicrobial agents [19].

#### Serotype analysis

The identification of capsular pneumococcal serotypes was performed at our laboratory using multiplex PCR combined with fragment analysis and automated fluorescent capillary electrophoresis. This technique implemented in our Molecular Microbiology Department in 2010 and allows the detection of 40 serotypes/serogroups: (1, 2, 3, 4, 5, 6A/6B, 6C, 7F/7A, 7C/(7B/40), 8, 9V/9A, 9N/9L, 10A, 10F/(10C/33C), 11A/11D/11F, 12F/(12A/44/46), 13, 14, 15A/15F, 15B/15C, 16F, 17F, 18/(18A/18B/18C/18F), 19A, 19F, 20, 21, 22F/22A, 23A, 23B, 23F, 24/(24A/24B/24F), 31, 33F/(33A/37), 34, 35A/(35C/42), 35B, 35F/47F, 38/25F, and 39) [20]. Pneumococcal strains were also sent to the National Center for Microbiology of Majadahonda (Madrid, Spain) to complete the serotype detection with Quellung reaction [21] and the antimicrobial susceptibility study.

#### Clonal analysis

Clonal analysis was performed with multilocus sequence typing (MSLT). MLST was performed as reported elsewhere [22]. The assignment of alleles and sequence types (ST) was carried out using the software at the pneumococcal web page http://pubmlst.org/spneumoniae/. Analysis of ST and assignment to clonal complex were performed with the eBURST program [23]. STs that shared six of seven allelic (single locus [SLV]) variants were considered a clonal complex.

### Statistical analysis

We used the *X*^2^ test or Fisher’s exact test to compare proportions. Statistical analyses were performed using SPSS for Windows, version 17.0 (SPSS). We calculated 95% CIs, and 2-sided *P* values < .05 were considered to be statistically significant.

Genetic diversity of isolate populations was estimated using Simpson’s numerical index of discrimination (SID), using the website: www.comparingpartitions.info. To compare the SID values from the different groups of data the *p* value was calculated using the online tool available in the same web page.

## Results

During the study period a total of 1576 pneumococcal invasive isolates were received, of which 25 were not viable for clonal analysis and serotyping. A total of 1551 strains (98.4%) were included in the study; serotype identification was performed in all of them. Among the 1551 isolates, 910 (58.7%) were from males and 641 (41.3%) from females, with a mean age of 50.7 years. 159 (10.2%) isolates were from children less than 2 years old, 143 (9.2%) from the age group between 2 to less than 5 years, 76 (4.9%) from the age 5 to less than 18 years, 561 (36.1%) from the age 18–65 and 612 (39.4%) in patients older than 65 years old.

All 1551 isolates of IPD had positive cultures: blood (1400), pleural fluid (78), cerebral spinal fluid (58), peritoneal fluid (2), joint fluid (5), ascites liquid (5) and pericardial fluid (1). In two of the positive cultures the sample type was not reported.

The most important clinical manifestation in the study period was pneumonia (*n* = 1078, 69.5%), including the diagnostics of non-complicated pneumonia (*n* = 980, 63.2%) and complicated pneumonia (*n* = 98, 6.3%), followed by bacteremia/sepsis (*n* = 315, 20.3%) and meningitis (*n* = 127, 8.2%). During the study period, although pneumonia stayed as the main clinical manifestation, a change in the proportion of the other diagnoses was observed. The proportion of meningitis and complicated pneumonia decreased from 44 cases (10.3%) and 29 cases (6.8%) in 2010 to 21 cases (6.1%) and 14 cases (4.1%) in 2013, with *p = 0*.*116* and *p = 0*.*110*, respectively. On the other hand, there was a significant increase in the proportion of cases with bacteremia/sepsis from 68 cases (15.9%) in 2010 to 86 cases (25.1%) in 2013, *p = 0*.*010*. According to age group, pneumonia was the main clinical manifestation diagnosed in all ages except in the group of children less than 2 years old where it was overcome by bacteremia/sepsis.

During the study period most of the *S*. *pneumoniae* invasive isolates were susceptible to the majority of the antibiotics analyzed. Penicillin (meningeal breakpoints), erythromycin and tetracycline presented a similar proportion of resistant isolates during the study period: 23.6%, 19.2% and 22.2%, respectively. Chloramphenicol, cefotaxime and levofloxacin showed lower proportions of resistance: 7%, 3% and 1%, respectively. However, three serotype 19A isolates (clonal type ST320) showed a MIC to cefotaxime ≥4 μg/ml. These strains were isolated in one child and two adults with pneumonia. No isolates were resistant to vancomycin. Overall, the proportion of multiresistance presented in the study was 13.2%. No significant differences in the rate of MDR were observed when comparing PCV13 serotypes vs non-PCV13 serotypes. Children less than 2 years old showed significantly higher proportion of MDR compared to older patients: 49 of 159 (30.8%) vs 156 of 1392 (11.2%) (*p<0*.*01*).

### Serotype distribution of invasive isolates

**Fig 1:**
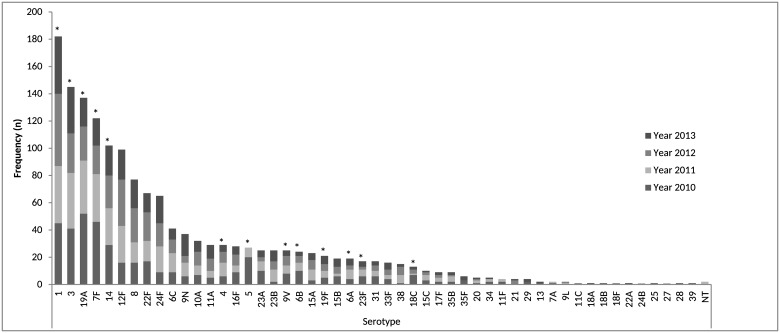
Frequency of serotypes causing invasive pneumococcal disease during the years 2010, 2011, 2012 and 2013.

Overall, 52 different serotypes were found. The most common serotypes found were serotype 1 (*n* = 182, 11.7%), serotype 3 (*n* = 145, 9.3%), serotype 19A (*n* = 137, 8.8%) and serotype 7F (*n* = 122, 7.9%) (Fig 1). In total, 14.9% were serotypes included in PCV7, 55.6% were serotypes included in PCV13 and 44.4% were non-vaccine serotypes.

According to age group, in children less than 2 years old the main serotypes were serotype 19A (*n* = 32, 20.1%), serotype 24F (*n* = 17, 10.7%) and serotype 14 (*n* = 15, 9.4%). In the age group between 2 and 5 years old, the most common serotype was serotype 1 (*n* = 48, 33.6%), as well as in the age group between 5 years old and 18 (*n* = 45, 59.2%). In the group between 18 years old and 65, the main serotypes were 7F (*n* = 62, 11.05%), 1 (*n* = 61, 10.9%) and 12F (*n* = 52, 9.3%). And in the age group older than 65 the main serotypes were serotypes 3 (*n* = 77, 12.6%), 19A (*n* = 60, 9.8%) and 14 (*n* = 45, 7.3%).

According to year, in 2010 the most common serotypes were serotype 19A (*n* = 52, 12.2%), serotype 7F (*n* = 46, 10.8%) and serotype 1 (*n* = 45, 10.5%). In 2011, the main serotypes found were serotypes 1 (*n* = 42, 10.2%), 3 (*n* = 41, 10%) and 19A (*n* = 39, 9.5%). In 2012, the most common serotypes were serotype 1 (*n* = 53, 14.3%), 12F (*n* = 34, 9.2%) and 3 (*n* = 29, 7.8%). And in 2013, they were serotypes 1 (*n* = 42, 12.2%), 3 (*n* = 34, 9.9%), 12F (*n* = 22, 6.4%) and 14 (*n* = 22, 6.4%).

During the study period there was an evolution in the serotypes included in the different conjugate vaccines. The serotypes included in the PCV7 maintained their proportion along the period of study, 16.6% in 2010 to 13.4% in 2013, whereas there was a significant proportional decrease in PCV13 serotypes from 65.3% in 2010 to 48.9% in 2013 (*p<0*.*01)*. Consequently, non-PCV13 serotypes showed a proportional significant increase, from 34.7% in 2010 to 51% in 2013 (*p<0*.*01)* (Table 1).

**Table 1:** Serotype distribution according to age group and year.

	PCV7(%)	PCV10(%)	PCV13(%)	Non PCV13(%)
**Age group**				
** <2 years old**	34 (21.4)	50 (31.4)	94 (59.1)	65 (40.9)
** 2 to <5 years old**	20 (14.0)	82 (57.3)	102 (71.3)	41 (28.7)
** 5 to <18 years old**	4 (5.3)	56 (73.7)	61 (80.3)	15 (19.7)
** 18 to 65 years old**	81 (14.4)	210 (37.4)	297 (52.9)	264 (47.1)
** >65 years old**	92 (15.0)	163 (26.6)	309 (50.5)	303 (49.5)
**Year**				
** 2010**	71 (16.6)	182 (42.6)	279 (65.3)	148 (34.7)
** 2011**	60 (14.6)	144 (35.1)	231 (56.3)	179 (43.7)
** 2012**	54 (14.6)	128 (34.5)	185 (49.9)	186 (50.1)
** 2013**	46 (13.4)	107 (31.2)	168 (48.9)	175 (51.0)

### Clonal diversity of serotypes

**Fig 2:**
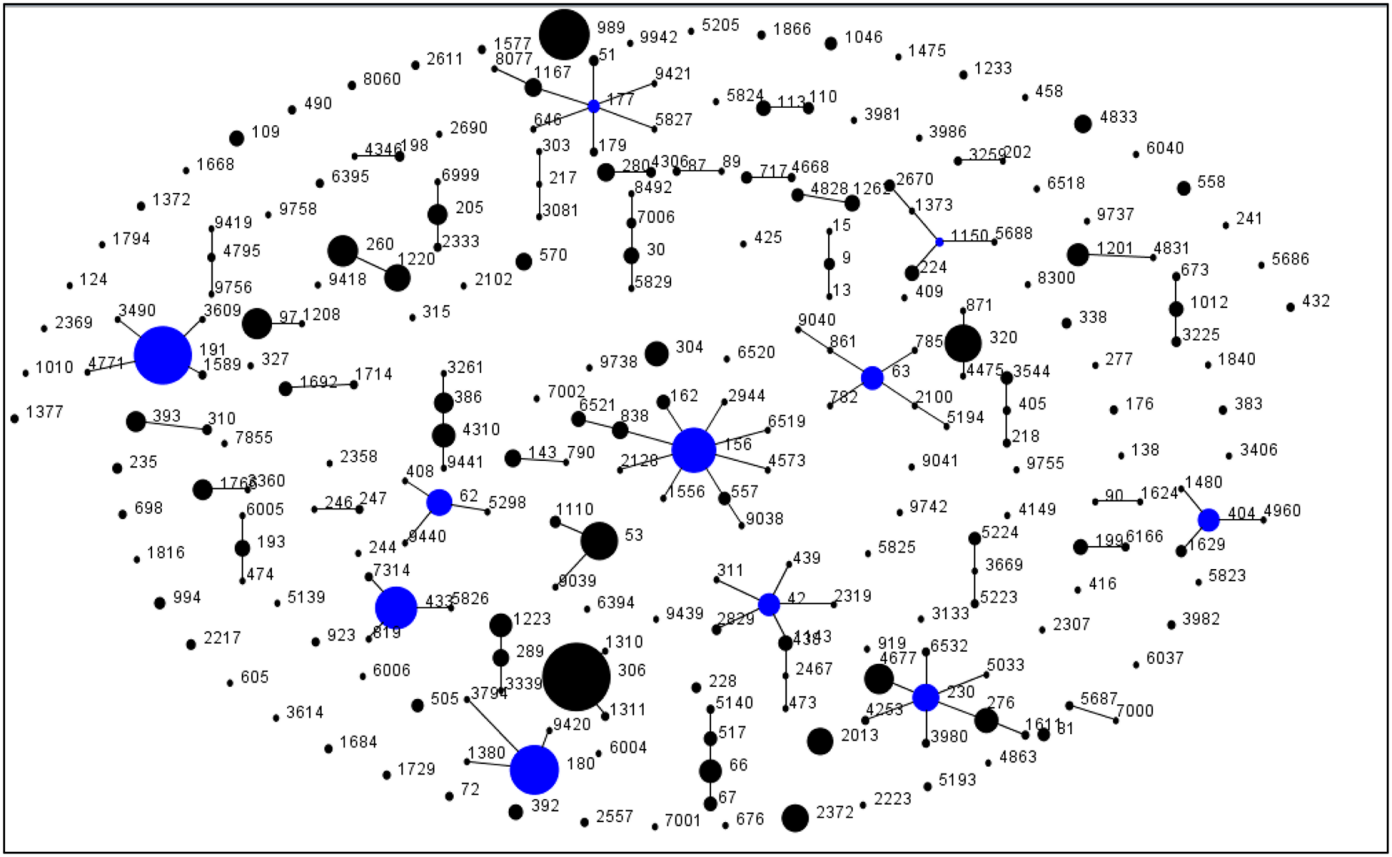
Clonal population of pneumococcal isolates obtained from 2010 to 2013 based on an eBURST analysis.

Overall, we identified 249 ST in the study, 45 of which were new (ST5223, ST5224, ST5686, ST5687, ST5688, ST5823, ST5824, ST5825, ST5826, ST5829, ST6004, ST6005, ST6006, ST6166, ST6394, ST6395, ST6518, ST6519, ST6520, ST6521, ST6999, ST7000, ST7001, ST7002, ST7314, ST7855, ST8077, ST8492, ST9038, ST9039, ST9040, ST9041, ST9419, ST9420, ST9421, ST9439, ST9440, ST9441, ST9737, ST9738, ST9742, ST9755, ST9756, ST9758, ST9942). Eight of these new STs also presented one new allele (ST8492, ST9439, ST9440, ST9441, ST9755, ST9756, ST9758 and ST9942). The most frequent clonal types found in the study were ST306 (*n* = 154, 9.9%), ST191 (*n* = 111, 7.2%), ST989 (*n* = 85, 5.5%) and ST180 (*n* = 80, 5.2%). A population snapshot of the 1551 isolates based on an eBURST analysis was represented in Fig 2. The major clonal complexes found were CC306, CC191, CC156, CC180, CC230, CC433, CC53 and CC320. Thirty-three STs expressed serotypes that were not present in the MLST database showing potential capsular switching (ST53, ST63, ST109, ST193, ST205, ST280, ST306, ST310, ST338, ST405, ST605, ST646, ST871, ST989, ST1046, ST1143, ST1167, ST1201, ST1816, ST2013, ST2217, ST2372, ST2557, ST2670, ST3133, ST4310, ST4475, ST4828, ST5033, ST5224, ST5688, ST6532 and ST8300).

Table 2 shows the different ST expressed by the different serotypes found in our collection, and the clonal diversity of each serotype was determined using the Simpson’s Index of Diversity (SID).

**Table 2:** Clonal diversity of serotypes causing IPD.

Serotype	SID^a^	CI (95%)	n°	CLONAL TYPES
**1**	0.283	0.200–0.367	182	217(1)/228(3)/303(1)/304(19)/306(153)/1310(1)/1311(2)/3081(1)/9942(1)
**3**	0.635	0.569–0.701	145	53(1)/180(79)/260(31)/458(1)/505(5)/1220(23)/1377(2)/1380(1)/3794(1)/9420(1)
**19A**	0.830	0.788–0.872	137	63(1)/162(1)/199(7)/202(1)/230(1)/276(19)/320(46)/416(1)/871(1)/994(4)/1167(6)/1201(15)/1611(2)/2013(22)/2102(1)/3259(2)/4475(1)/4831(1)/6005(1)/6166(2)/8077(1)/9742(1)
**7F**	0.201	0.104–0.298	122	191(109)/393(1)/1589(2)/2372(1)/3133(1)/3490(1)/3544(5)/3609(1)/4771(1)
**14**	0.586	0.472–0.700	102	9(4)/13(1)/15(1)/63(1)/124(1)/143(9)/156(65)/162(1)/409(1)/433(1)/557(5)/782(1)/790(1)/1556(1)/2128(1)/2944(1)/4573(1)/5193(2)/6519(1)/6520(1)/7002(1)/9038(1)
**12F**	0.272	0.160–0.384	99	218(2)/989(84)/2013(1)/4833(10)/8060(2)
**8**	0.631	0.525–0.736	77	53(44)/404(16)/989(1)/1110(4)/1480(1)/1629(4)/3225(3)/3406(1)/4960(1)/5205(1)/9039(1)
**22F**	0.251	0.111–0.391	67	433(58)/698(2)/819(1)/1372(2)/2557(1)/5826(1)/7314(2)
**24F**	0.690	0.612–0.769	65	72(2)/162(1)/177(3)/230(22)/646(1)/3980(2)/4253(2)/4677(29)/5033(1)/6532(2)
**6C**	0.779	0.667–0.892	41	224(6)/386(2)/1143(1)/1150(1)/1692(5)/1714(2)/2670(2)/3261(1)/4310(18)/5688(1)/9418(1)/9441(1)
**9N**	0.772	0.662–0.881	37	66(16)/67(6)/280(1)/405(1)/517(6)/3982(2)/5140(2)/5824(1)/6004(1)/6037(1)
**10A**	0.123	1.000–0.278	32	97(30)/1208(1)/5686(1)
**4**	0.825	0.693–0.957	29	205(12)/244(1)/246(1)/247(2)/1729(2)/1866(2)/2333(2)/2358(1)/4795(2)/6006(1)/6999(1)/9419(1)/9756(1)
**11A**	0.571	0.401–0.741	29	62(18)/408(1)/1010(1)/5298(1)/6521(7)/9440(1)
**16F**	0.817	0.729–0.906	28	30(8)/383(2)/570(9)/1840(1)/5829(1)/6395(2)/7006(3)/8492(1)/9758(1)
**5**	0.510	0.382–0.638	27	289(9)/1223(17)/3339(1)
**23A**	0.587	0.369–0.804	25	42(16)/438(1)/2319(1)/2670(2)/2829(3)/5825(1)/9439(1)
**23B**	0.157	1.000–0.348	25	1373(1)/2372(23)/7001(1)
**9V**	0.740	0.649–0.831	25	156(1)/162(3)/280(9)/838(9)/4306(3)
**6B**	0.793	0.629–0.958	24	90(1)/138(1)/176(2)/315(1)/386(11)/1624(1)/1668(1)/2467(1)/3614(1)/5687(2)/6394(1)/7000(1)
**15A**	0.640	0.410–0.870	23	63(14)/193(1)/605(1)/861(1)/1262(1)/1577(1)/5139(1)/5224(1)/7856(1)/9041(1)
**19F**	0.943	0.896–0.990	21	51(3)/87(2)/89(1)/177(2)/179(2)/180(1)/425(1)/1167(4)/2100(1)/2307(1)/5194(1)/5827(1)/9421(1)
**15B**	0.912	0.851–0.974	19	193(2)/474(1)/1262(4)/1577(1)/3669(1)/4828(4)/5223(2)/5224(2)/8300(1)/9040(1)
**6A**	0.901	0.794–1.000	19	224(1)/327(1)/338(1)/473(1)/490(2)/919(1)/1143(6)/1150(1)/1692(1)/2611(2)/3981(1)/3986(1)
**31**	0.419	0.140–0.698	17	1684(2)/1766(13)/3360(1)/7855(1)
**23F**	0.765	0.631–0.898	17	81(5)/109(7)/277(1)/311(1)/338(2)/439(1)
**33F**	0.758	0.617–0.900	16	673(2)/717(4)/1012(7)/2223(1)/4668(2)
**38**	0.385	0.092–0.678	14	310(2)/393(11)/1201(1)
**18C**	0.718	0.570–0.866	13	110(4)/113(6)/205(1)/1233(2)
**15C**	0.844	0.743–0.946	10	63(1)/193(3)/1262(3)/4828(1)/5224(2)
**17F**	0.417	0.047–0.786	9	310(1)/392(7)/9738(1)
**35B**	0.750	0.579–0.921	9	198(3)/558(4)/2690(1)/4346(1)
**35F**	0.933	0.805–1.000	6	676(1)/2217(2)/6518(1)/9737(1)/9755(1)
**34**	0.000	0.000–0.000	5	1046
**20**	0.700	0.349–1.000	5	235(3)/1794(1)/2369(1)
**29**	0.833	0.583–1.000	4	1816(1)/558(2)/2217(1)
**11F**	0.000	0.000–0.000	4	62
**21**	0.667	0.667–0.667	4	193(2)/432(2)
**13**	0.000	0.000–0.000	2	923
**7A**	0.000	0.000–0.000	2	191
**9L**	1.000	1.000–1.000	2	66(1)/405(1)
**25**	1.000	1.000–1.000	1	393
**27**	1.000	1.000–1.000	1	1475
**28**	1.000	1.000–1.000	1	5823
**24B**	1.000	1.000–1.000	1	230
**11C**	1.000	1.000–1.000	1	53
**39**	1.000	1.000–1.000	1	6040
**18A**	1.000	1.000–1.000	1	241
**18B**	1.000	1.000–1.000	1	113
**18F**	1.000	1.000–1.000	1	4863
**22A**	1.000	1.000–1.000	1	2557
**NT**			2	
**Total**			1551	

^a^ SID, Simpson’s Index of Diversity. Years 2010, 2011, 2012 and 2013.

## Discussion

The results obtained in this study allowed us to observe the evolution experienced by serotypes after the introduction of PCV13 in our geographical area. After the introduction of the vaccine and, similarly to other countries with higher vaccine coverage, there has been a significant proportional decrease in the PCV13 serotypes and, as a consequence, a proportional increase of non-PCV13 serotypes [24–28]. However, PCV13 serotypes continue to be responsible for a high burden of the disease, probably due to the low vaccine coverage which is estimated in only 40% of target population in our geographical area [13]. Despite this limited coverage, serotypes 19A and 7F experienced an important decrease during the study period; a similar decrease was not observed for serotypes 1, 3 and 14. In the non-vaccine serotypes group, it is worth mentioning the increase observed for serotype 12F along the study period, and compared with previous data obtained before the introduction of PCV13 in our region [29]. This increase was also noticed in the post PCV13 studies of Israel, Germany and Uruguay previously cited [24,26,28]. In our study, a low index of diversity of serotype 12F was observed, with high proportion of ST989, a clonal type previously detected in children with meningitis in Gambia [30]. Also interesting, serotype 24F was the second most common serotype in children less than 2 years old during the study period when analyzing the results by age groups. The major clone detected in serotype 24F was ST230, a multidrug resistant clone related with the increase of serotype 19A in Bulgaria, Portugal and in our geographical area [29,31,32]. A previous study conducted in our area also observed an increase in the rate of serotype 24 several years after PCV7 introduction associated with the clonal expansion of this genotype [33].

The clonal analysis of serotype 19A revealed the important presence of the multiresistant clone ST320 in 46 of 137 (33.5%) serotype 19A isolates, but a high index of clonal diversity was found which reflects the high adaptation of this serotype. The association of serotype 19A with ST320 was previously described in our area as a contributor to the emergence of this serotype after the introduction of PCV7 [34]. The high proportion of ST320 among our isolates (three of them showing high resistance to cefotaxime) is worrisome as well as the still high presence of this serotype despite being included in PCV13, which highlights the need for complete vaccination to prevent disease caused by this serotype.

A worrying result is the proportionally high prevalence of serotype 14 found in our results. This serotype was included in PCV7, the first generation of conjugate vaccines available in Spain since 2001. While other PCV7 serotypes were almost non-existent in our study, serotype 14 continues to be one of the most prevalent in younger children and older people. Its clonal composition revealed that ST156 was detected in more than 65 of 102 (63%) serotype 14 isolates. A previous study performed by our group found that the pilus was present in all ST156 strains analyzed, while its general presence in the other clonal types was not frequent [35]. A study performed by Barocchi et al. showed in a murine model that a strain with pilus was more virulent than its non-piliated mutant [36]. Moreover, a study by Sjöström et al. suggested that the expression of pili in ST156 isolates contributed to the successful spread of this clone worldwide [37]. Although undoubtedly the low coverage of conjugate vaccines in our population could be facilitating its spread, we hypothesize that the high presence of this virulence factor in the serotype 14 isolates of our geographical area could be also related to the persistence of its circulation and their high invasiveness. Further studies are needed to know why serotype 14 continues to be so prevalent in our population.

Also noteworthy were the results about serotype 3. The present study confirms the results obtained for this serotype in previous publications of our group ranking as the second serotype more frequently found causing IPD. In our previous studies serotype 3 was also found responsible of a high burden of disease in our area when detection and capsular typing was performed in direct sample by molecular techniques [38–40]. These results differ from the ones previously obtained from Brueggemann et al. in England where serotype 3 was only found in carriage [41]. Contradictory results have also been found with respect the clonal diversity of this serotype. Studies from Scotland and Singapore described a high clonal diversity for this serotype, while studies from Canada and The Netherlands found strong clonal homogeneity of serotype 3 in their countries [42–45]. In our study a low index of diversity was found with CC180, which comprises ST180 and three SLV (ST3794, ST1380 and ST9420), being the major clonal type detected among serotype 3 isolates in our geographical area (82 of 145 isolates, 56.5%). In contrast, ST180 was associated with a small number of IPD cases in a pediatric study performed in the US during the 2008–2013 period [46].

### Conclusion

In conclusion, despite the observed decrease of PCV13 serotypes, these serotypes continue to be the major serotypes causing invasive disease in our geographical area. These results emphasize the need for complete PCV13 vaccination in children to cover the main affected population and also to ensure the protection obtained by herd immunity. The study of the genetic population of the serotypes circulating in a geographical area might help to obtain useful information about temporal trends, geographical differences and changes in invasiveness.
